# The order of things: phosphate release or the power stroke, which does actomyosin do first?

**DOI:** 10.3389/fphys.2025.1692606

**Published:** 2025-11-26

**Authors:** Edward P. Debold, Christopher P. Marang, Brent D. Scott

**Affiliations:** 1 Department of Kinesiology, University of Massachusetts, Amherst, MA, United States; 2 Department of Chemical Engineering, Stanford University, Palo Alto, CA, United States; 3 Department of Health Sciences, College of William and Mary, Williamsburg, VA, United States

**Keywords:** myosin, power stroke, phosphate, force generation, actin

## Abstract

Myosins are a highly conserved super family of motor proteins that are responsible for driving a host of intracellular processes in eukaryotes, from muscle contraction to vesicular transport. Myosins can perform these tasks because they transduce chemical energy, from the hydrolysis of ATP into mechanical work, in the form of a power stroke. The key event in the transduction process is the putative coupling of P_i_ release with the power stroke; however, the timing and mechanism of coupling of these events remain unclear. Atomic structures of myosin, captured in intermediate states of its cross-bridge cycle, suggest that P_i_ release is required for the power stroke to occur and therefore must precede the power stroke. In contrast, most functional assays, which can measure myosin’s structural dynamics with sub-millisecond temporal and nanometer spatial resolution, suggest that the power stroke occurs less than 1 ms after forming a strong bond with actin, while P_i_ release occurs 10–200 ms after binding to actin, suggesting that the power stroke precedes P_i_ release. A host of new studies and a few new models have been put forth in recent years to attempt to reconcile these seemingly conflicting findings. Although there is not yet a consensus on the order of these events, the new information provided by these efforts is transforming our understanding of how myosin transduces energy. This knowledge has important implications for elucidating the molecular basis of a myriad of myosin-associated diseases and, therefore, for the development of compounds to treat these diseases.

## Introduction

**FIGURE 1:**
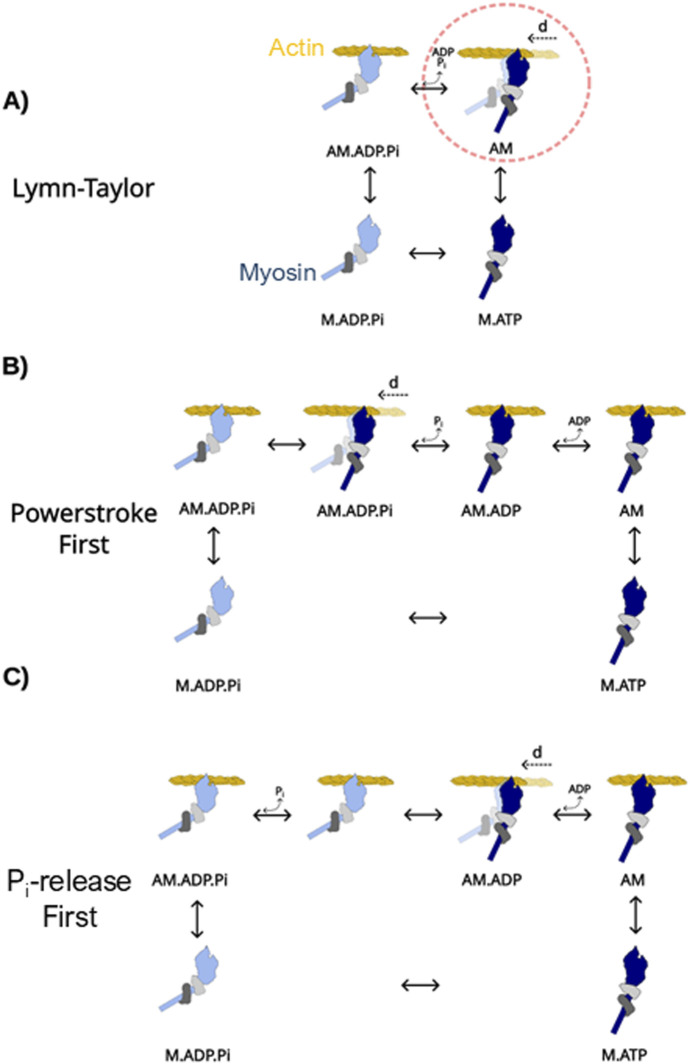
Models of the cross-bridge cycle. Myosin (blue) is shown as a myosin II motor with two light chain domains (light and dark gray). The power stroke denoted by “d” occurs when bound to the actin filament (yellow). The power stroke is denoted by myosin’s lever arm changing from a light blue (pre-power stroke) to dark blue (post-power stroke). **(A)** The four-state model of the cross-bridge cycle, originally proposed by Lymn & Taylor in 1971. The power stroke (circled in red) occurs with the release of P_i_ and ADP in this model. **(B)** A power stroke-first model, in which myosin rapidly generates a power stroke upon strongly binding to actin prior to the release of P_i_ from the nucleotide-binding site. **(C)** A P_i_-release-first model in which strong binding to actin rapidly triggers the release of P_i_ from the nucleotide-binding site.

Myosins are a superfamily of ATPases that are responsible for driving various forms of cellular motility, from cytokinesis to vesicular transport (Vale, 2000; Vale, 2003). They can drive these processes because they transduce the chemical energy, from ATP hydrolysis, into force and/or motion as they undergo cyclic interaction with an actin filament. The key event in the transduction process is the 60–70° rotation of a long alpha helical coil or lever arm (i.e., power stroke) that is putatively coupled to the release of gamma P_i_ following the hydrolysis of ATP (Geeves and Holmes, 1999). Thus, if these events are indeed coupled, this would link the mechanical power stroke to the release of a product of hydrolysis. Support for tight coupling between these events was provided by estimates that suggested that the largest decrease in free energy occurred with the release of P_i_ (Howard, 2001; Inoue et al., 1989). Based on this, and related findings, the first models of myosin’s cross-bridge cycle (Figure 1) often showed product release occurring concomitantly with the formation of a strong bond to the actin filament (Bagshaw and Trentham, 1974; Chock et al., 1979; Lymn and Taylor, 1971). However, subsequent experiments suggested that although these events may be coupled, they do not appear to occur simultaneously (Takagi et al., 2004). Indeed, the formation of the strongly bound state with actin appears to precede both the power stroke and the release of P_i_ (Dantzig et al., 1992; Sun et al., 2008; Takagi et al., 2004). A vigorous debate over which event occurs first exists, the power stroke or P_i_ release from the active site (Debold, 2021; Hwang et al., 2021; Kawai, 2025; Llinas et al., 2015; Månsson et al., 2023; Moretto et al., 2022; Muretta et al., 2015; Robert-Paganin et al., 2020).

It is also unclear how key structural elements of myosin’s active site might coordinate its motions to couple actin binding with P_i_ release and the power stroke (Llinas et al., 2015; Preller and Holmes, 2013; Robert-Paganin et al., 2020). Gaining an understanding of the order of events and the structural dynamics involved in this process is important not only for understanding the fundamental basis of energy transduction by motor enzymes but also for guiding the design of drugs to alter the force-generating capacity of myosin to treat diseases such as heart failure (Hei et al., 2024; Planelles-Herrero et al., 2017; Woody et al., 2018). Given this importance, there has been a significant effort to reveal these mechanisms, and recent advances in cryo-electron microscopy (Zhang and Liu, 2018) and biophysical assays (Capitanio et al., 2012; Muretta et al., 2015) are providing opportunities to shed light on these events with nanometer spatial and millisecond precision. In this review, we summarize some of the early research on this question to provide historical contexts before detailing some of the most significant findings from recent studies.

### The power stroke and P_i_ release occur rapidly after myosin strongly binds to actin

Myosin couples the generation of force and/or motion to the hydrolysis of ATP and the release of its products ADP and P_i_ in a series of steps known as the cross-bridge cycle (Lymn and Taylor, 1971). It was postulated early in the burgeoning field of muscle biophysics that the mechanical transitions might alternate with biochemical transitions (Huxley, 1974). Subsequent transient kinetic experiments led to the proposal that some of the mechanical transitions are tightly coupled with the biochemical transitions (Bagshaw and Trentham, 1974). An early version of the cross-bridge cycle (Figure 1) included steps in which ATP binding to myosin rapidly induces detachment from actin in a post-power stroke state (Geeves and Holmes, 2005), after which hydrolysis occurs, and the lever arm is re-primed to a pre-power stroke position (Steffen and Sleep, 2004). ADP and P_i_ are released at a slow rate, while myosin is detached from actin (Lymn and Taylor, 1971), but when myosin forms a strong, stereo-specific bond with the actin filament, it accelerates both the release of P_i_ from the active site and the generation of the power stroke (Bagshaw and Trentham, 1974; Lymn and Taylor, 1971), which represents the large rotation of a long alpha helix or lever arm within the motor domain (Holmes and Geeves, 2000; Rayment et al., 1993b). These events are then followed by the release of ADP, which is thought to be closely associated with (Jacobs et al., 2011) or even coupled (Capitanio et al., 2006; Veigel et al., 2003) to a second smaller rotation of the lever arm (Houdusse et al., 1999). Once these mentioned steps are complete, myosin is again in the rigor state, and if the ATP concentration is high, the cycle would rapidly repeat. A key aspect of the cycle, established in the 1970s, was that upon strongly binding to actin, both the power stroke and the release of P_i_ occur very rapidly (Bagshaw and Trentham, 1974; Lymn and Taylor, 1971), presenting a challenge to resolving these events in detail because they were beyond the time resolution of most functional assays used at that time.

Information from crystal structures of myosin suggest that the power stroke is initiated by small conformational changes in the nucleotide-binding site (also known as the active site) that become amplified in the converter domain to cause the 60–70° rotation of the lever arm (Geeves and Holmes, 2005; Preller and Holmes, 2013; Rayment et al., 1993a; Sweeney and Houdusse, 2010). The conformational changes in the active site are thought to be induced by the formation of a strong bond with the actin filament, which also induces conformational changes that accelerate the release of P_i_ from the active site (Geeves and Holmes, 2005; Holmes and Geeves, 2000; Robert-Paganin et al., 2020; Sweeney and Houdusse, 2010). Thus, these three events are thought to be coupled such that the conformational changes that occur at the actin-binding site are rapidly communicated to the nucleotide-binding site and then amplified in the converter domain, causing rotation of the lever arm (i.e., a power stroke) once myosin has established a strong bond with the actin filament (Geeves and Holmes, 1999). This hypothesized communication pathway provides a structural basis for the coupling of these events. However, technological developments in functional assays have suggested that these events may not occur simultaneously and thus may not be perfectly coupled, with some suggesting that the power stroke may precede the release of P_i_ from the active site (Gunther et al., 2020; Marang et al., 2023; Marang et al., 2025; Muretta et al., 2015; Scott et al., 2021; Trivedi et al., 2015; Woody et al., 2019), while others have suggested the opposite sequence of events (Llinas et al., 2015; Moretto et al., 2022; Robert-Paganin et al., 2020). Some researchers have also suggested that these events may not be tightly coupled, positing that the power stroke does not depend on the presence of P_i_ in the catalytic site and that P_i_ can be released in any biochemical state (Caremani et al., 2013).

These observations, which suggest that these events occur at different times and rates, led to an obvious question of the relative timing of each event, i.e., which event happens first? In particular, do the conformational changes induced by strong binding to the actin filament facilitate the release of P_i_ or the power stroke first? Moreover, does one event “drive” or “gate” the other, for example, does P_i_ release “gate” the power stroke (Llinas et al., 2015) or does the power stroke “gate” the release of P_i_ (Trivedi et al., 2015)? The answer to this question is essential to understanding the molecular mechanisms underlying myosin’s ability to transduce chemical energy into mechanical work. Simplified versions of the two competing models of the cross-bridge cycle are shown in Figure 1.

A related question, important for understanding why force is depressed in muscle during muscle fatigue (Allen, 2009; Debold et al., 2016; Fitts, 1994) or myocardial ischemia (Allen and Orchard, 1987), is what happens to myosin when P_i_ rebinds to actomyosin with ADP still in the active site. Some data suggest that the power stroke is reversed before myosin rapidly detaches from actin (Dantzig et al., 1992; Hwang et al., 2021; Woody et al., 2019). However, this can occur only if the power stroke precedes P_i_ release (Takagi et al., 2004); if P_i_ release occurs first, then rebinding of P_i_ would prevent rather than reverse the power stroke (Robert-Paganin et al., 2020; Sweeney and Houdusse, 2010). There is also evidence to suggest that the re-binding of P_i_ may induce myosin to detach without reversing the power stroke (Debold et al., 2013; Marang et al., 2025). This idea was inspired by, but is distinct from, the hypothesis stated by Caremani et al. (2008) and the model of Linari et al. (2010), which suggested that P_i_ rebinding induces detachment from a strongly bound state to actin following a reversal of the power stroke, but myosin remains in a structural state with an accelerated rate of P_i_ release. Thus, these questions remain unanswered but are essential for understanding the mechanisms of the loss of force in muscle fatigue and myocardial ischemia. The answers will be critical to the development of drugs attempting to modulate the force- and motion-generating capacity of myosins to treat a myriad of diseases, including various forms of muscle weakness and heart failure, because it is essential to identify which step of the cross-bridge cycle to target to purposefully enhance or inhibit the force- and motion-generating capacity of myosin (Auguin et al., 2024; Lam et al., 2023; Scott et al., 2024; Woody et al., 2018).

Despite the importance of this knowledge, it has been difficult to determine the relative timing of P_i_ release and the power stroke because of the following reasons: 1) it is difficult to generate high-resolution structures of the actomyosin complex in intermediate states of the cross-bridge cycle (Llinas et al., 2015; Preller and Holmes, 2013); and 2) the power stroke is thought to occur <1 ms after myosin strongly binds to the actin filament (Capitanio et al., 2012; Dunn and Spudich, 2007), which is faster than most methods of detection in standard functional assays. However, recent advances in cryo-electron microscopy (Zhang and Liu, 2018) and single-molecule biophysical techniques are pushing the spatial and temporal resolution toward values required to answer these questions (Gunther et al., 2020; Llinas et al., 2015; Muretta et al., 2015; Trivedi et al., 2015; Woody et al., 2019).

### Early functional assays suggested that the power stroke preceded P_i_ release

The questions regarding the relative timing of P_i_ release and the power stroke emerged from the effort to understand the molecular basis of force generation during muscle contraction (Huxley, 1957; 1974; Huxley and Niedergerke, 1954; Huxley and Simmons, 1971; Huxley, 1969; Huxley and Hanson, 1954). As mentioned above, transient kinetic experiments on isolated muscle proteins demonstrated that actin greatly accelerates the release of P_i_ and ADP from the active site (Bagshaw and Trentham, 1974; Lymn and Taylor, 1971). Combining these data with early negative stain electron micrographs of muscle sarcomeres (Huxley, 1963; Huxley, 1967; Huxley, 1969; Reedy et al., 1965) led to the first proposed cross-bridge cycle, in which the power stroke was coupled to product release, both P_i_ and ADP (Lymn and Taylor, 1971). Subsequent solution experiments indicated that P_i_ is released before ADP and that P_i_ release is coupled to the largest decrease in Gibbs free energy, suggesting that this step is irreversible (Bagshaw and Trentham, 1974; Inoue et al., 1989). However, these experiments were performed in the absence of load and without direct measures of the power stroke; thus, it was unclear how these events were coupled to force generation and movement of the actin filament during contraction. To address this aspect, researchers performed experiments on chemically skinned single muscle fibers, where force can be directly measured while controlling the composition of the intracellular solutions (Cooke and Pate, 1985; Hibberd et al., 1985). These findings suggested that P_i_ release and force generation were not irreversible, provided the cross-bridge was under a resistive strain, such as during a maximal isometric contraction (Cooke and Pate, 1985; Hibberd et al., 1985). Caged phosphate compounds were used to overcome the diffusion limitations across the muscle fiber, allowing for the nearly instantaneous and uniform release of millimolar amounts of P_i_ during a maximal isometric contraction (Dantzig et al., 1992). These experiments showed clearly that force was not reduced immediately after the release of caged phosphate, as would be predicted if these events were perfectly coupled. There was a concentration-dependent delay of a few milliseconds before the force began to decrease, and the rate of force decay was dependent on the P_i_ concentration (Dantzig et al., 1992). These observations were corroborated and extended by others (Homsher et al., 1997), including elegant experiments using single myofibrils, in which diffusion limitations are also overcome (Tesi et al., 2000). These observations were shown to be consistent with a model in which myosin first strongly-binds to actin in a pre-force-generating, pre-power stroke AM.ADP.P_i_ state before generating force through a power stroke and subsequently releasing P_i_. Moreover, these events are then reversible when the cross bridge experiences a resistive strain, if P_i_ is readily available in the surrounding solution. These experiments also suggested that the actomyosin state, to which P_i_ rebound, is the first of two ADP-bound states, separated by an isomerization (Sleep and Hutton, 1980) occurred before actomyosin reaches the rigor state. The first ADP-bound state (AM.ADP) is load-sensitive, such that it is prolonged by a resistive load and accelerated by an assistive load (Kad et al., 2007; Veigel et al., 2003; Veigel et al., 2005). P_i_ is thought to only rebind to the AM.ADP state prior to isomerization, but after much of the power stroke is completed (Hibberd et al., 1985; Webb et al., 1986). However, a caveat of these experiments was that the rate of P_i_ release was not directly measured but was inferred from the changes in isometric force to the increase in P_i_ in solution.

The subsequent development of a phosphate-binding protein that could rapidly detect P_i_ in solution enabled the simultaneous measurement of force and P_i_ release (He et al., 1997). Experiments on single muscle fibers using this reagent revealed that isometric force develops earlier and at a faster rate than that of P_i_ release into the myoplasm (He et al., 1997; Sleep et al., 2005). Thus, these and similar findings provided compelling evidence that the power stroke precedes the release of P_i_ (Stehle and Tesi, 2017). However, these early approaches could not provide direct observations of myosin’s power stroke or directly visualize when and how P_i_ was exiting the nucleotide-binding pocket. This level of detail could be provided by high-resolution crystal structures of myosin.

#### Crystal structures of myosin suggest that P_i_ release gates the power stroke

A related question, being pursued based on high-resolution crystal structures of myosin, is which structural elements and conformational changes are associated with the power stroke and P_i_ release in the effort to understand the molecular basis of force generation by myosin. The first atomic structure of myosin, using fast skeletal myosin II, was crystallized in the absence of ATP, demonstrating the lever arm in a post-power stroke configuration (Rayment et al., 1993b). The 2.8 Å resolution of crystals provided a detailed description of the key elements of the nucleotide-binding site responsible for its enzymatic activity, including the highly conserved switch I, switch II, and P-loop elements. Under these rigor-like conditions, switch II was in an “open” state, a position not likely to make contact the gamma phosphate of ATP and thus is not a state capable of hydrolyzing ATP. This was interpreted as an indication of a low affinity for P_i_, and thus, this configuration would putatively facilitate the release of P_i_ from the nucleotide-binding site or active site.

The other obvious feature in the structure was a large open cleft that splits the actin-binding domain, made up of the upper and lower 50-KDa domain. When this structure was fitted into the envelop of decorated actin (with myosin bound to actin in rigor) generated from lower-resolution cryo-EM images, the best fit was obtained when this actin-binding cleft from the atomic structure was closed to match the dimensions of the actomyosin interface in the cryo-EM structures (Rayment et al., 1993a). The authors astutely hypothesized that when myosin forms a strong bond with actin, it induces the closure of this actin-binding cleft. Combining the two observations, they suggested that the closure of the actin-binding cleft is coupled to the motions of switch II, such that cleft closure reduces myosin’s affinity for P_i_, while an open cleft allows myosin to tightly bind to the gamma phosphate of ATP. Consistent with this notion, when the actin-binding cleft is open, a highly conserved glycine residue of switch II makes contacts with the gamma P_i_, enabling hydrolysis of ATP, while this contact is lost in the rigor state (Geeves and Holmes, 2005). This idea provides a structural basis for myosin’s reciprocal affinity for actin and the gamma phosphate of ATP. Combining these observations with the fact that the lever arm is in a post-power stroke state led to the hypothesis that these events were coupled. In addition, the authors speculated that P_i_ must exit the active site, through a “backdoor” (Yount et al., 1995), for the power stroke to occur, suggesting that P_i_ release gates the power stroke and thus must occur first (Sweeney and Houdusse, 2010).

Subsequent structures were generated in the presence of various analogs of ATP to directly visualize the contacts between the nucleotide and the key elements of the active site. *Dictyostelium* disodium myosin II was crystallized with MgADP and either beryllium or aluminum fluoride in the active site to mimic the ATP-bound state and transition state of hydrolysis, respectively (Fisher et al., 1995; Reubold et al., 2003; Smith and Rayment, 1996). In these structures, switch II has multiple residues within the hydrogen bonding distance of the gamma phosphate of ATP, indicating that it is in a hydrolytically competent state. This position of switch II is now known as the “closed” position (Fisher et al., 1995). Switch II is also in this “closed” position in the presence of MgADP and vanadate (an analog of P_i_) (Smith and Rayment, 1996). Although the active site is in this configuration, the converter region and the truncated version of the light chain-binding domain were observed in positions consistent with the lever arm being in a primed or pre-power stroke state (Reubold et al., 2003). This suggests that when P_i_ is in the nucleotide-binding site, in an M.ADP.P_i_ biochemical state, the lever arm remains in a pre-power stroke position. The motions of switch II are thought to be tightly coupled to the rotation of the lever arm through the “relay helix,” which connects switch II to the converter domain, where these motions are amplified to produce the large rotation of the lever arm (Geeves and Holmes, 2005). By contrast, when myosin was crystallized under conditions mimicking the ADP-bound or rigor states, the converter and/or lever arm adopted a configuration consistent with the post-power stroke state (Coureux et al., 2003; Houdusse et al., 1999; Rayment et al., 1993b). These observations provided additional, albeit indirect, support for the notion that P_i_ release putatively gates the power stroke. However, these findings are based on static images of myosin obtained in the absence of actin, and it is known that actin induces changes in the structure of myosin (Behrmann et al., 2012; Klebl et al., 2025) that are not captured even in the presence of analogs of ATP that mimic transition states. In addition, it is also important to note that, in solution, the structural elements of the active site and the other subdomains fluctuate among extreme positions, especially in the transition states (Conibear et al., 2003; Málnási-Csizmadia et al., 2001; Ostap et al., 1995; Yengo et al., 2002). Thus, to capture these motions, researchers have leveraged the increased resolution provided by the significant advances in cryo-EM in the last 10 years (Zhang and Liu, 2018) and have used techniques to track myosin’s structural dynamics with millisecond (Gunther et al., 2020), and, in some cases, microsecond (Capitanio et al., 2012; Woody et al., 2019) time resolution. Below we highlight some of the results that have taken advantage of these technological developments to provide key new insights into the questions on the nature of these crucial processes in myosin’s transduction mechanism.

### Capturing the structure of the P_i_-release state

**FIGURE 2:**
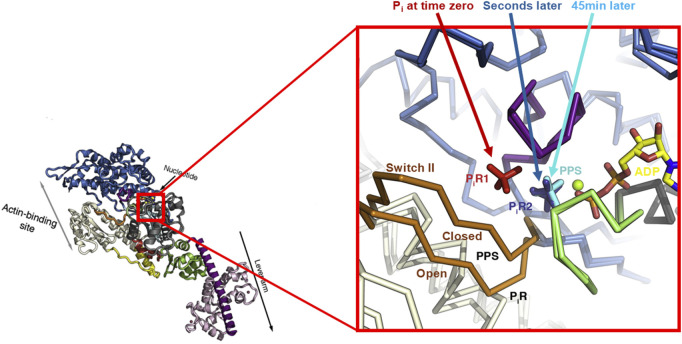
Proposed P_i_-release pathway from myosin’s nucleotide-binding site. A) Crystal structure of myosin VI with the nucleotide-binding site enlarged (right). The position of P_i_ is depicted in the pre-power stroke state (PPS) and in the two P_i_-release states, P_i_R2 and P_i_R1. The P_i_R1 structure was obtained immediately after (time zero) soaking myosin VI with ADP in a high [P_i_] buffer; the P_i_R2 state was obtained after seconds of exposure to high P_i_ and the PPS after 45 min of soaking. In the PPS state, P_i_ is inside the active site, switch II is in the “closed” position, and the converter is in a pre-power stroke configuration (not shown). With P_i_ in the P_i_R2 state, P_i_ has moved slightly outside of the active site, and switch II in now in an “open” position. In the P_i_R1 state, P_i_ has moved further from the active site, and switch II remains open. The converter is in a post-power stroke configuration in both the P_i_R2 and P_i_R1 states (not shown). Figure modified from Llinas et al. (2015) with permission from the publisher.

A key missing piece of structural information was a detailed description of the P_i_-release state. To capture this state, Llinas et al. (2015) developed a novel procedure to crystallize myosin in the presence of elevated levels of P_i_. Crystals of the truncated motor domain of myosin VI were grown in the presence of MgADP and a cryo-protectant containing 25–100 mM P_i_. Myosin VI was mixed with this solution and quickly frozen at different time points after exposure to the high levels of P_i_ to capture P_i_ at different points in the release/rebinding process. Samples frozen immediately after exposure to the P_i_-containing solution resulted in a structure where P_i_ was near the exit of the putative P_i_-release tunnel, dubbed the first phosphate-release state (P_i_R1), but the converter region remained in a post-power stroke configuration. Allowing myosin VI to remain in the P_i_ buffer for a few seconds before freezing resulted in a second phosphate-release structure (P_i_R2), in which P_i_ was located near ADP but not fully within the nucleotide-binding region (Figure 2). Importantly, in this structure, switch II has moved 4 Å, opening the putative tunnel to allow for P_i_ to be released. However, switch I and the P-loop remain unchanged, consistent with findings from solution kinetics showing that P_i_ is released, while the MgADP is still bound in the nucleotide-binding site (Bagshaw and Trentham, 1974). This truncated construct did not have a lever arm, making it difficult to predict its position; however, the converter domain was in a configuration similar to its position in the post-power stroke state. Therefore, the authors hypothesized that these states (P_i_R2 and P_i_R1) are the states from which P_i_ is released from the nucleotide-binding site.

Allowing rigor myosin with MgADP to be exposed to the high P_i_ solution for 45 min generated a structure in which P_i_ made it all the way back into the nucleotide-binding pocket adjacent to MgADP (Figure 2). In this structure, termed the PPS or pre-power stroke state, the converter region adopted a pre-power stroke configuration, and switch II had moved back into a closed position, blocking P_i_’s exit from the active site. Thus, these conditions allowed the myosin, which was started in a rigor configuration with MgADP, to reform the pre-power stroke state with ADP and P_i_ in the active site.

Assuming that the rebinding of P_i_ follows the same path as its release, as there may be multiple pathways (Cecchini et al., 2010), these structures putatively reveal the pathway of phosphate’s exit from the nucleotide-binding site to the point it would be released into the solution, suggesting that P_i_ is released from the active site into a tunnel, where a few amino acid side chains are capable of making contacts with the exiting P_i_, slowing its entry into the solution (Figure 2). To examine this, several myosin constructs were made with mutations along the putative P_i_-release tunnel to determine whether they would affect the rate of P_i_ release. The first was a mutation in switch I (S217A, in myosin Va), which was hypothesized to impede P_i_ from entering the release tunnel, effectively slowing its exit from the nucleotide-binding pocket. Previous work proposed that the loss of the hydroxyl group with the removal of serine, which putatively contacts the gamma phosphate of ATP, would impede the entry of P_i_ into the P_i_-release tunnel (Forgacs et al., 2009). Consistent with this hypothesis, the presence of this mutation reduced the P_i_-release rate from 143/s to only 38/s in the myosin Va construct, providing at least indirect evidence that P_i_ release was impeded from the active site (Llinas et al., 2015). This finding was also observed in other classes of myosin in this study, and others have reported more dramatic reductions in the rate of P_i_ release with the S217A construct in myosin Va (Forgacs et al., 2009; Gunther et al., 2020), confirming its role in slowing P_i_ release from the active site.

Subsequently, mutations were introduced inside the putative P_i_-release tunnel to either increase or decrease the bulkiness or charge in the region (Llinas et al., 2015). Consistent with the hypothesis, adding amino acids with bulky side chains to the tunnel slowed P_i_ release, and introducing amino acids that reduced the bulkiness or decreased the charge in the tunnel either accelerated or did not affect the rate of P_i_ release. This series of structures and kinetic findings led the authors to hypothesize that P_i_ is released from the nucleotide-binding site very rapidly and before the power stroke, but its release into the solution is slowed by the charged residues within the P_i_-release tunnel, delaying its appearance in the solution (Llinas et al., 2015). This was a very novel idea because it provided an explanation for the observation that force development in muscle fibers precedes the appearance of P_i_ in solution (He et al., 1997) and provided support for the idea that P_i_ is released before the largest portion of the power stroke.

### P_i_ release before the power stroke challenges conventional models of the cross-bridge cycle

However, having P_i_ release occur before the power stroke challenges the widely accepted models of the cross-bridge cycle and the observations from single-muscle fibers from which they were developed (Pate and Cooke, 1989a; Takagi et al., 2004). For example, if the power stroke, and thus force-generation, occurs prior to P_i_ release, then exposing single-muscle fibers to elevated levels of P_i_ should not cause large reductions in force, but it is well established that the isometric force in the muscle is demonstrably decreased by millimolar levels of P_i_ (Cooke and Pate, 1985; Debold et al., 2004; Hibberd et al., 1985; Tesi et al., 2000). Similarly, elevated levels of P_i_ would be expected to slow the contraction velocity if P_i_ is released before the power stroke, but it is also well established that P_i_ has little or no effect on unloaded shortening velocity (Debold et al., 2004; Pate and Cooke, 1989b) and can even increase the velocity at elevated levels of ATP (Pate and Cooke, 1989b) and at low pH (Debold et al., 2011; 2012). A key issue for the latter observation is that the measured rate of P_i_ release is slower than that of ADP release, which limits contraction velocity in muscle fibers (Nyitrai et al., 2006) and actin filament velocity in an *in vitro* motility assay (Tyska and Warshaw, 2002). This model would also seem to be inconsistent with the observation that elevated levels of P_i_ depress force more than the ATPase activity (Bowater and Sleep, 1988; Potma et al., 1995), which has been successfully modeled assuming a power stroke-first model (Linari et al., 2010; Marang et al., 2025).

However, the effects of P_i_ on isometric force, shortening velocity, and some mechanical transients in muscle fibers can be reproduced using a P_i_-release-first model if the rate of P_i_-release is increased to 3,000/s, in a more complex model of the cross-bridge cycle (Moretto et al., 2022). However, it remains unclear whether such a model can fit data showing that the mechanical transients are independent of [P_i_] (Caremani et al., 2013). Thus, it may be possible to reconfigure the kinetics and the cross-bridge model to capture the effects of P_i_ on the contractile properties of muscle fibers, but in the current form, it requires the rates of P_i_ release that are far faster than the reported values in solution (Muretta et al., 2015; Tang et al., 2021; Trivedi et al., 2015).

A second caveat with the new structure, revealing the putative P_i_-release tunnel (Llinas et al., 2015), is that it was obtained using myosin VI, which has structural features that are not present in the remaining members of the myosin superfamily. However, Moretto et al. (2022) provided evidence that P_i_ might also bind to alternative sites on muscle myosin II using total internal reflectance fluorescence (TIRF) microscopy to perform a competition assay with fluorescent ATP. In particular, muscle myosin II was exposed to high concentrations of fluorescently labeled ATP, and then, it was competed off by exposure to increasing concentrations of unlabeled P_i_. The fluorescent spots decreased in a pattern that was dependent on the P_i_ concentration. Although the resolution of these TIRF experiments did not indicate precisely where on the myosin surface P_i_ was binding, parallel experiments using blebbistatin and vanadate led the authors to conclude that P_i_ binds to myosin at sites other than the nucleotide-binding site (Moretto et al., 2022).

This same paper reported findings from a high-speed atomic force microscope, which was used to image actomyosin in various nucleotide states (Moretto et al., 2022). The spatial resolution of this technique is much lower than that of X-ray crystallography or cryo-EM, particularly for determining the position of myosin’s lever arm; however, using coordinates from atomic models of myosin, they reported observing myosin in an ADP-bound state in both pre- and post-power stroke states, suggesting that the power stroke can reverse in the absence of P_i_. If this finding is correct, it would support the idea that P_i_ is released before the power stroke and that these events are only loosely coupled (Caremani et al., 2013).

To gain more direct insights into the force-generating steps of myosin on actin, a research group took advantage of the recent advances in cryo-EM using a highly innovative technique to capture the structure of actomyosin in the pre-power stroke and post-power stroke states (Klebl et al., 2025). They rapidly froze myosin in an ADP.P_i_ state after mixing it with actin and imaged the samples using cryo-EM at two time points; 10 ms and 120 ms after mixing myosin and actin. They used a double-mutant myosin Va construct with a 10-fold reduced rate of P_i_-release and an increased affinity for actin to ensure that the bound state lasted long enough to capture myosin bound to the actin filament. The findings revealed that myosin was in two distinct populations when bound to actin, with the lever arm in either a pre-power stroke or a post-power stroke state, and the proportion of these populations shifted based on the time after fixation on the EM grids. In the pre-power stroke configuration, there were hydrophobic interactions between the lower 50-KDa domain of myosin and actin, but the salt bridge that putatively prevents P_i_ release (Holmes and Geeves, 2000; Sweeney and Houdusse, 2010) remained intact, indicating that P_i_ would not be released in this configuration (the P_i_ atom was not resolved in these structures). In addition, the actin-binding cleft is not completely closed, which, in the absence of load, is tightly coupled to lever arm rotation (Geeves and Holmes, 2005). This suggests that the actomyosin state captured is prior to either P_i_-release or the generation of the power stroke.

In the post-power stroke configuration, which represented the dominant actomyosin population at 120 ms, the actin-binding cleft is fully closed, and the salt bridge between switch I and switch II is broken, suggesting that P_i_ has been released. Therefore, this represents an actomyosin structure after both the power stroke and P_i_ release have occurred.

This was a ground-breaking study designed to definitively demonstrate support for the swinging lever arm mechanism of force generation by myosin (Rayment et al., 1993a; Tyska and Warshaw, 2002) and provide some of the most definitive evidence for the rotation of the lever arm. The findings also confirmed that the power stroke and P_i_ release occur close in time, but it was not designed to resolve which event occurs first. Repeating these experiments with the goal of capturing the structures at intermediate time points and with higher spatial resolution to visualize P_i_ in the active site may provide novel insights into the timing and mechanisms of P_i_ release and the power stroke. For example, it would be exciting to capture an actomyosin structure with P_i_ outside the active site; however, with myosin still in a pre-power stroke state, this would be supportive of P_i_ release preceding the power stroke (Llinas et al., 2015). Alternatively, a post-power stroke structure with P_i_ still in the active site would support the idea that the power stroke precedes P_i_ release (Gunther et al., 2020; Muretta et al., 2015; Scott et al., 2021; Trivedi et al., 2015). Indeed, such a structure is predicted to exist based on findings from molecular dynamics simulations of the initial steps of the force generation (Preller and Holmes, 2013). With further advances in cryo-EM techniques (Zhang and Liu, 2018), these structures could be revealed in the near future.

### Direct measures of the structural dynamics and rates of biochemical reactions

Seizing upon the technological advances in functional assays, researchers have used various biophysical techniques to answer the question of the relative timing of P_i_ release and the power stroke in fully functioning myosin and actin.

The development of fluorescence resonance energy transfer (FRET)-probes for different regions of myosin has enabled researchers to directly observe myosin’s structural dynamics with millisecond time-resolution in solution, including the timing of closure of the actin-binding cleft (Sun et al., 2008) and measurement of the rate of the power stroke relative to the rate of the P_i_ release into solution (Muretta et al., 2015).

The Yengo Laboratory used this technology to examine the relative timing of the closure of the actin-binding cleft and the rate of P_i_ release from actomyosin (Sun et al., 2008). Their findings suggested that myosin Va, when in an M.ADP.P_i_ state, rapidly binds to actin and that the actin-binding cleft closes immediately upon the formation of a strong bond with the actin filament. They also showed that this rate was much faster than that of P_i_ release into solution. In addition, they concluded that the nucleotide-binding pocket was still closed immediately after the actin-binding cleft had closed, suggesting that these events might not be perfectly coupled. This finding is important because the closure of the actin-binding cleft is thought to be tightly coupled to the rotation of myosin’s lever arm (Geeves and Holmes, 2005; Rayment et al., 1993a); therefore, this suggested that the power stroke occurs in the strongly bound state prior to P_i_ release. However, the position of the lever arm was not directly monitored in this study and would require further developments to the FRET system to determine its position.

The Thomas Laboratory developed a technique to directly observe these changes over the course of an experiment using time-resolved FRET (TR^2^-FRET). With a fluorescent probe on myosin’s regulatory light chain, serving as the donor and a fluorescently labeled nucleotide acting as the acceptor of the FRET pairing, this provided a precise measure of the distance between the probes in a transient kinetic assay (Muretta et al., 2015). Taking advantage of a stopped-flow device, myosin was pre-incubated with ATP for a few minutes, placing it in an M.ADP.P_i_ state, before it was rapidly mixed with actin, at which point they observed the rate of the transition from the pre- to post-power stroke state of the lever arm. Using the time-resolved version of this assay allowed for the determination of the average rate of myosin transitioning from the pre- to post-power stroke state with nanometer spatial and millisecond temporal resolution. In a parallel set of experiments, they measured the rate of P_i_ release under the same conditions using the stopped-flow device (Muretta et al., 2015).

**FIGURE 3:**
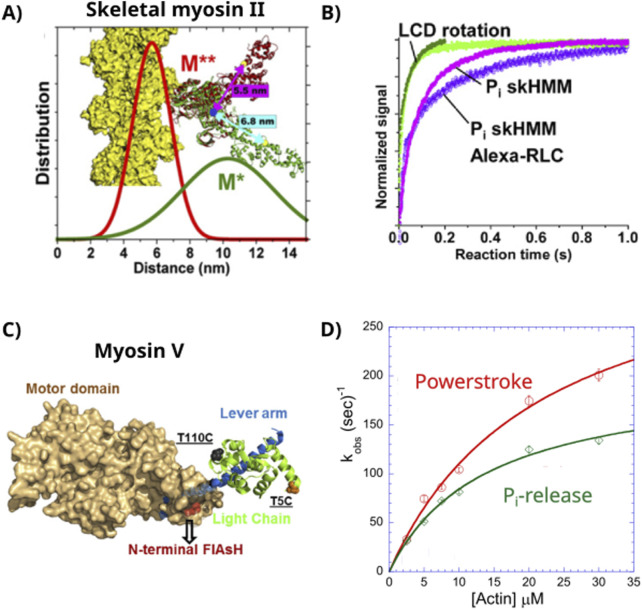
FRET and stopped-flow experiments. **(A)** The skeletal myosin II construct (skHMM) used to measure the rate of the power stroke using TR_2_FRET. **(B)** The rate of the rotation of the light chain-binding domain (LCD), i.e., the power stroke, after exposure to actin (green traces). **(B)** The rate of P_i_ release following mixing of myosin in an ADP.P_i_ state with actin (purple traces). The power stroke occurred at a rate of ∼350/s, while P_i_-release reached a maximum of ∼25/s. **(C)** Structure of the myosin Va construct used in FRET experiments to measure the rates of the lever arm rotation and P_i_ release after mixing with actin in a stopped-flow device. **(D)** Rate of each reaction plotted as a function of the actin concentration. The extrapolated maximum rate of the lever arm rotation was 352/s, and the rate of P_i_ release was ∼200/s. Figures modified from Muretta et al. (2015) and Trivedi et al. (2015) with permission from the publisher.

With the fast skeletal muscle myosin II (HMM) used in this study, the FRET signal revealed two rates of lever arm rotation. The fast rate of the power stroke was consistent with the large rotation of myosin’s light chain domain (lever arm) prior to ADP release, and the slower rate was consistent with the mechanical events following ADP release. This was consistent with prior observations showing that myosin generates a large power stroke that is temporally associated with P_i_ release and a smaller sub-step or hitch near the release of ADP (Houdusse et al., 1999; Veigel et al., 2003). The fast rate was dependent on the actin concentration and reached a maximum of >350/s. In contrast, the rate of P_i_ release saturated at a much slower rate of ∼25/s (Figure 3). Thus, these findings suggested that the power stroke occurs at a rate that is more than 10 times faster than the release of P_i_. The authors were careful to acknowledge that the finding of the faster rate of the power stroke alone does not require it to precede P_i_ release; therefore, they modeled the results to help delineate between a power stroke- or P_i_-release-first model. These simulations revealed that the data were very poorly fitted using a P_i_-release-first model but were well fitted using a power stroke-first model (Muretta et al., 2015). This suggests that once strongly bound to actin, myosin rapidly generates a power stroke before releasing P_i_ at a much slower rate.

The Yengo Laboratory made similar measurements using a single-headed myosin Va construct (Figure 3) and came to the same conclusion (Trivedi et al., 2015). This was an important finding because myosin Va is known to have an actin-activated rate of P_i_ release that is almost 10-fold faster than that of fast skeletal muscle myosin II (Baker et al., 2004; Cruz et al., 1999; Rosenfeld and Sweeney, 2004). Indeed, they estimated the rate of P_i_ release to be ∼200/s in the single-headed construct used in the study; however, the FRET-based assays showed that the rotation of the lever arm (power stroke) reached a maximum rate of 500/s at saturating actin concentrations, more than 2-fold faster than P_i_ release in the same construct under the same conditions. Thus, assuming that when myosin forms a strong bond with actin, both events are accelerated simultaneously, the power stroke would occur prior to the release of P_i_ from the active site, at least into solution. Indeed, they also performed computer simulations to help delineate between the power stroke-first and P_i_-release-first mechanisms and found that their data were most consistent with a mechanism where the power stroke occurs prior to P_i_ release. Thus, the FRET-based experiments that can measure the structural dynamics of the power stroke and the rate of P_i_-release in solution suggest that the power stroke occurs very rapidly following the formation of a strong bond with actin, and then, P_i_ is released more slowly into solution.

However, these findings and the associated model simulations did not incorporate the idea that P_i_ may be released from the active site but stall in the putative release tunnel, as suggested by Kawai (2025), Llinas et al. (2015), and Moretto et al. (2022). Therefore, it is possible that in these experiments, P_i_ moved out of the active site faster than it appeared in the solution. The findings from the FRET experiments would, however, put time constraints on the details of a P_i_-release-first mechanism. For example, for muscle myosin II, the release of P_i_ from the active site would have to occur at faster than 350/s and then stall in the P_i_-tunnel for several milliseconds before appearing in solution to match the observed rate of 25/s. Moreover, the rates for the power stroke obtained in the FRET-based assays did not appear to saturate (Figure 3), suggesting that the power stroke may occur even faster than 350/s, putting even stricter time constraints on the P_i_-release-first model.

### Findings from laser trap assays

Advances in the single-molecule laser trap assay have provided researchers with even greater time resolution to resolve the power stroke. This assay was first used to directly determine the size of the power stroke generated by a single myosin molecule and the duration of a single actomyosin binding event (Finer et al., 1994). Using a three-bead version of the laser trap assay, the Spudich Laboratory showed that skeletal muscle myosin rapidly generates a 10–12 nm power stroke faster than the resolution of the assay ∼2–10 ms (Finer et al., 1994), which is limited by the corner frequency of the 1-micron-diameter microsphere diffusing in water (∼500 Hz) (Guilford et al., 1997). Later, the Spudich Laboratory showed, using dark field microscopy, that myosin’s power stroke was indeed faster than a few milliseconds, occurring at a rate of several hundred microseconds under the unloaded conditions of the assay (Dunn and Spudich, 2007).

**FIGURE 4:**
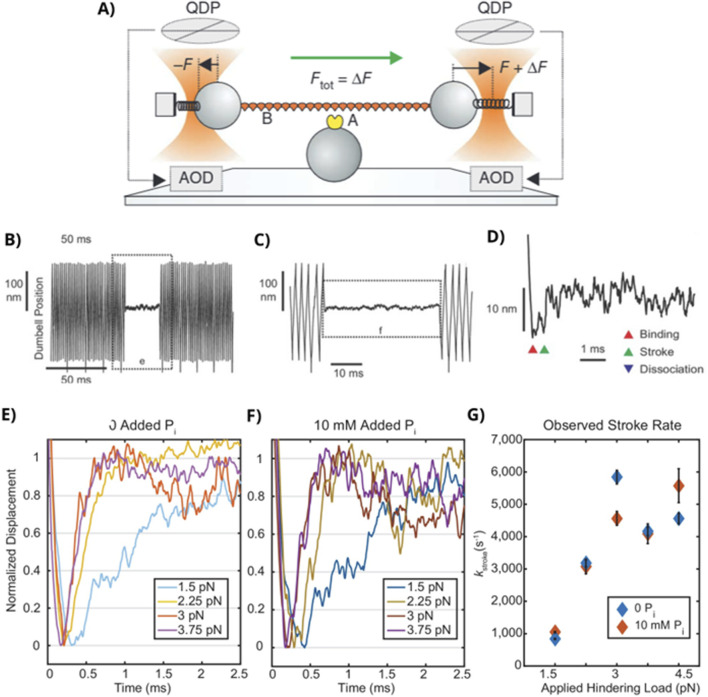
Ultra-fast laser trapping assay supports a power stroke-first model. **(A)** Schematic representation of the ultra-fast laser trap assay, originally described by Capitanio et al. (2012). Reprinted with permission. The bead–actin–bead assembly is oscillated in a sawtooth wave pattern using two feedback systems to apply a constant force to the actin filament. **(B)** Woody et al. (2019) used a similar assay to detect the binding of myosin to actin as a pause in the oscillations. During a binding event, the load is clamped at a prespecified level until myosin detaches and the oscillations resume. **(C)** An expanded view of panel **(B)**. **(D)** An expanded time scale of panel **(C)** indicating the initial binding of myosin to actin (red triangle), the power stroke (green triangle), and detachment of myosin from actin, purple triangle (not shown). **(E)** Ensemble averages of the start of the binding events in the absence and **(F)** presence of 10 mM P_i_. **(G)** The rate of the power stroke, taken from fits to ensemble averages, plotted vs. the magnitude of the hindering or resistive load. Figures modified from Woody et al. (2019) with permission from the publishers.

In a groundbreaking advance of this assay, Capitanio et al. (2012) increased the time resolution by almost an order of magnitude by actively oscillating the bead–actin–bead assembly in a sawtooth pattern over the pedestal with myosin on the surface (Figure 4). By driving the motion of the bead–actin–bead assembly, the time resolution is no longer limited by the thermal motion of the optically trapped bead and can be improved to ∼100 µs, which is faster than the predicted rate of the power stroke (Dunn and Spudich, 2007). Using single-headed fast skeletal myosin II, they observed two populations of binding events: those that lasted <1.5 ms, which did not generate a displacement, and events lasting >1.5 ms which generated a 4–5 nm power stroke (Capitanio et al., 2012). The short events were likely weak interactions between myosin and actin that did not generate a power stroke. By contrast, three distinct mechanical events were present in the events lasting >1.5 ms; 1) the initial binding to actin putatively in a pre-power stroke state, followed by 2) a displacement of the actin filament of 4–5 nm, i.e., the power stroke, and 3) detachment from actin. Importantly, ensemble averaging of the binding events, including the initial binding and subsequent power stroke, was completed within 1–2 ms. Therefore, this would stipulate that the actin-activated rate of P_i_ release from myosin’s active site would have to occur at a rate greater than 500–1,000/s to precede the power stroke.

A similar instrument was used by Woody et al. (2019) in an experiment specifically designed to address the relative timing between P_i_ release and the power stroke, using a two-headed fragment (HMM) of cardiac muscle myosin II. They determined the size and rate of the power stroke in the absence and presence of 10 mM P_i_. In the absence of added P_i_, they also observed two populations of binding events, with the short events less likely to generate a step, particularly at higher resistive forces. In addition, the size of the power stroke decreased at increasing resistive load, but the rate of the power stroke increased with the resistive load, from 700/s at 1.5 pN to more than 5,000/s at 3 pN (Figure 4). The increased rate of the power stroke was thought to occur because the duration of time spent in the pre-power stroke state decreases with the magnitude of the resistive load, leaving only the binding events that rapidly transition through the power stroke (Woody et al., 2019). Thus, this finding is likely not in conflict with the observations in muscle fibers, showing that the working stroke becomes slower with a resistive load (Reconditi et al., 2004).

Most importantly, 10 mM P_i_ had no effect on either the magnitude of the power stroke or the rate at which it occurred (Figure 4). Moreover, these force values are within the range of 3–6 pN predicted to be generated by myosin in isotonic and isometrically contracting muscle (Piazzesi et al., 2007). If correct, these findings would put even stricter time constraints on a model, in which P_i_ release occurs prior to the power stroke. In particular, to be consistent with a P_i_-release-first model, P_i_ would need to be released at rates >5,000/s from the active site and then need to pause in the P_i_-tunnel for more than 100 ms to match the observed release rate of 8–10/s for beta cardiac myosin (Tang et al., 2021). This scenario seemed unlikely; therefore, the authors presented a model in which the power stroke preceded P_i_ release, which was able to reproduce their findings (Woody et al., 2019).

An interesting additional finding from this study was that the number of short- and intermediate-duration binding events without a power stroke increased in the presence of elevated P_i_ (Woody et al., 2019), but this occurred only under the lowest resistive forces (1.5 and 2.25 pN). The authors attributed this to an increase in the frequency of detachment from a weakly bound state, implying that neither the power stroke nor P_i_ release occurred during these events. An alternative explanation (Robert-Paganin et al., 2020) suggested that this increase was the result of P_i_ rebinding to actomyosin in an ADP-bound state prior to the generation of the power stroke. Thus, P_i_ would have been released, but the power stroke was prevented because a new P_i_ from solution rebound to myosin’s active site while still in an ADP-bound state, reforming an ADP.P_i_ state from which it detached. The second explanation would be consistent with P_i_ release preceding and gating the power stroke (Llinas et al., 2015).

Another challenge to the interpretation of the findings from Woody et al. (2019) is that the experiments in the presence of P_i_ were conducted at an ATP concentration of 1 µM, suggesting that the bound lifetime included a significant amount of time in the rigor state. Although P_i_ readily rebinds to actomyosin in the AM.ADP state, it can also compete with ATP for binding to the rigor state, forming an off-pathway AM.P_i_ state that putatively prolongs rigor, until ATP successfully binds to the active site (Amrute-Nayak et al., 2008; Debold et al., 2011). Indeed, this effect may be the reason for the 2-fold reduction in the detachment rates observed in the presence of P_i_ in this study (Woody et al., 2019).

Another version of the laser trap assay was used (Hwang et al., 2021) to study the effects of ADP and P_i_ on the mechanics of a single cross-bridge. In this study, single-muscle myosin molecules were initially attached to an actin filament under high resistive load (7–15 pN) in rigor conditions (no ATP), after which ADP and subsequently P_i_ were added to the experimental buffer. Using cardiac muscle myosin, they observed small reversals of the power stroke, but only in the presence of ADP; once P_i_ was added, no reversals were observed. These observations led them to conclude that the first power stroke occurs after P_i_ is released from the active site, and a second sub-step occurs with ADP release. However, the prior literature has suggested that there are multiple actomyosin–ADP states, with some suggesting that the state formed when ADP rebinds to the rigor state may constituent an off-pathway state (Hannemann et al., 2005; Rosenfeld et al., 2000; Siemankowski and White, 1984). It would be interesting to determine which ADP state or states were being probed in this investigation and which of these P_i_ rebound to.

### Testing the P_i_-release-first model

**FIGURE 5:**
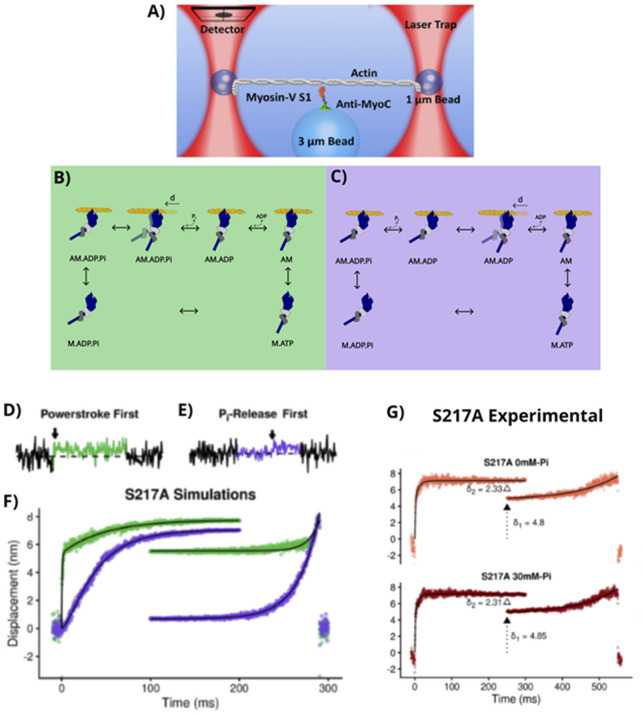
Testing the P_i_-release-first hypothesis. **(A)** A schematic representation of the three-bead laser trap assay used by Scott et al. (2021). **(B)** The power stroke-first cross-bridge model (green background) and **(C)** the P_i_-release first model (purple background). Pre-power stroke states are shown in light blue, same as in Figure 1. Simulations of displacement vs. time from single actomyosin-binding events based on either **(D)** a power stroke-first or **(E)** a P_i_-release-first model using the reaction rates determined from stopped-flow measurements of the S217A myosin Va construct reported by Gunther et al. (2020). Dotted lines drawn to show baseline, i.e., zero displacement. **(F)** Forward and backward ensemble averages of simulated actomyosin-binding events for the S217A construct using either a power stroke-first (green) or P_i_-release-first model (purple). **(G)** Forward and backward ensemble averages of the experimental data with the S217A myosin Va construct. Values shown report the size of the main power stroke in nm (δ_1_) and the second sub-step or hitch (δ_2_), also in nm. The experimental data are most consistent with a power stroke-first model. Figures modified from Scott et al. (2021) with permission from Wiley Publishers Inc.

The proposal of the existence of a P_i_ tunnel (Llinas et al., 2015) presented a testable hypothesis for researchers to directly test in functional assays. A central premise of P_i_-gated power stroke theory (Llinas et al., 2015; Sweeney and Houdusse, 2010) is that when P_i_ is held in the nucleotide-binding pocket, the lever arm is prevented from rotating. Therefore, if P_i_ could be prevented from leaving the nucleotide-binding pocket, the power stroke should be prevented, or at least delayed, until the P_i_ finally leaves the active site. This idea was tested using FRET-based and single-molecule laser trap assays (Gunther et al., 2020; Scott et al., 2021). The first approach to keep P_i_ in the active site utilized a mutation in switch I (S217A), which is thought to impede the release of P_i_ from the nucleotide-binding site into the release tunnel (Forgacs et al., 2009; Llinas et al., 2015). Two studies examined the effect of this mutation of myosin’s ability to bind and translocate an actin filament in the single-molecule laser trap assay and a FRET-based experiment described above (Gunther et al., 2020; Scott et al., 2021). They first confirmed, using transient kinetic methods, that the S217A mutation dramatically slowed the actin-activated rate of P_i_ release (Gunther et al., 2020), consistent with the effect hypothesized by Llinas et al. (2015). Importantly, both studies found that the presence of this mutation had no effect on either the size of the power stroke or the rate at which myosin transitioned from a pre- to post-power stroke state (Gunther et al., 2020; Scott et al., 2021). Scott et al. (2021) tried a second, independent, approach to maintaining P_i_ in the active site by elevating P_i_ to 30 mM concentration in the experimental solution in a laser trap assay (Figure 5). Similar to the findings with the S217A mutation, the elevated levels of P_i_ did not alter the magnitude nor the rate of the power stroke at the single-molecule level. This occurred despite evidence that P_i_ was indeed rebinding to actomyosin in an ADP-bound state, for example, a reduction in the lifetimes of the longest binding events in the presence of P_i_ (Scott et al., 2021). A later study that increased the resistive load on the myosin observed that P_i_ caused even greater reductions in the lifetime of bound events, but P_i_ still did not affect the size and rate of the power stroke (Marang et al., 2023), suggesting that the power stroke was occurring with P_i_ still in the nucleotide-binding pocket.

To determine which model was favored, Scott et al. (2021) simulated data from a single-molecule laser trap assay based on either a power stroke-first model or a P_i_-release-first model (Figure 5). Using published values of the P_i_-release rate from the S217A construct in solution, Forgacs et al. (2009), Gunther et al. (2020), and Llinas et al. (2015) demonstrated that if P_i_-release gated the power stroke, the single-molecule laser trap assay would have detected binding events in which myosin bound in a strongly bound pre-power stroke state before transitioning to a post-power stroke state within the same binding event (Figure 5). However, they observed no evidence of these kind of binding events either in their raw data or using the more sensitive ensemble averaging analysis (Scott et al., 2021). Therefore, they believed that their data were most consistent with the power stroke preceding the release of P_i_ from the active site.

**FIGURE 6:**
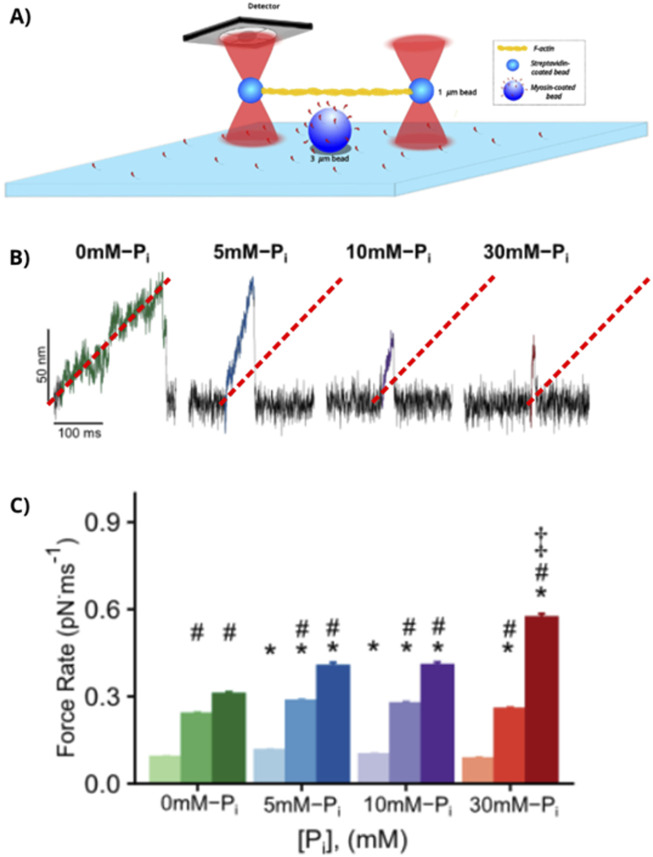
Effect of force and P_i_ on myosin’s force-generating capacity. **(A)** A schematic representation of the mini-ensemble laser trap assay, in which multiple myosin molecules bind to and translocate the actin filament. **(B)** Raw displacement records at a laser trap stiffness of 0.20 pN/nm in the presence of increasing concentrations of P_i_. The dashed read line depicts the slope of the trace in the absence of added P_i_ and is superimposed on the other [P_i_] levels to show that the rate of force development increased as [P_i_] was increased. **(C)** Mean ± SEM rates of force development, with darker shading depicting laser trap stiffness increasing from 0.04 to 0.20 pN/nm. * indicates significantly (p < 0.05) different from 0 mM-P_i_) at each laser trap stiffness. # indicates different from 0.04 pN/nm trap stiffness at the same [P_i_]. ‡ indicates different from all [P_i_] at the same laser trap stiffness. Figures modified from Marang et al. (2025) with permission from the publishers.

Although this work was performed in myosin Va (Scott et al., 2021), the same group has recently come to a similar conclusion based on findings from fast skeletal muscle myosin II (Marang et al., 2025). The duration of ADP lifetime of fast skeletal muscle myosin [∼2 ms; Nyitrai et al. (2006)] is beyond the resolution of a standard three-bead laser trap assay; therefore, to ensure constant contact with the actin filament, these experiments were performed with mini-ensembles of myosin and at 100 µM ATP. A unique aspect of this study was that they varied the amount of resistive load on the myosin and the concentration of P_i_ (Figure 6). They observed that the resistive load increased the lifetime of binding events in the absence of P_i_, consistent with load-dependent ADP release (Kad et al., 2007; Veigel et al., 2003; Veigel et al., 2005). However, the binding event lifetimes were dramatically reduced once P_i_ was introduced, and the effect was most pronounced at the highest P_i_ and resistive loads (Marang et al., 2025). Strikingly, the rate of force development of the binding events increased in the presence of P_i_, which was consistent with a model in which the power stroke precedes P_i_ release from the active site (Figure 6). Thus, based on these functional data from the laser trap assay, it appears that the behaviors at least of myosin Va and of fast skeletal muscle myosin II are most consistent with the power stroke-first model.

The observations that the power stroke occurs rapidly upon the formation of a strong actomyosin bond (Scott et al., 2021; Woody et al., 2019) and is independent of the concentration of P_i_ (Marang et al., 2025) seem to be consistent with the findings from skinned muscle fibers, suggesting that working stroke occurs rapidly upon myosin binding to actin (Reconditi et al., 2004) and is unaffected by the [P_i_] (Caremani et al., 2013). This suggests that myosin’s working stroke is driven not by the release of P_i_, as originally proposed (Bagshaw and Trentham, 1974; Howard, 2001; Inoue et al., 1989), but by the closure of the actin-binding cleft, which allows for the formation of the strong bond with actin, an idea that has been proposed previously (Karatzaferi et al., 2004).

## Conclusion

The mechanism of force transduction by myosin is an important aspect of motor enzymes and highly relevant to the development of pharmaceutical compounds to modulate myosin function in a myriad of cells. The key events in the transduction of chemical energy into mechanical work are the release of P_i_ and the large rotation of the lever arm. Although they were originally thought to be tightly coupled events and occur simultaneously, recent evidence points toward them occurring at different times and rates. High-resolution crystal structures of myosin have led authors to suggest that P_i_ release occurs first and gates the power stroke, but it is possible that a post-power stroke state with P_i_ still in the active site exists but has yet to be captured. Studies on functional proteins, leveraging advances in biophysical techniques, have generally revealed data that favor models where the power stroke precedes P_i_ release (Gunther et al., 2020; Marang et al., 2023; Marang et al., 2025; Muretta et al., 2015; Scott et al., 2021; Trivedi et al., 2015; Woody et al., 2019).

The next major advance in structural information may come from the new technique for capturing actomyosin states using the cryo-EM technique, developed by Howard White et al. (Klebl et al., 2025), in which a transient state is observed with P_i_ still bound to the active site after completion of the power stroke, or with P_i_ released into the tunnel and the lever arm in a post-power stroke state. Parallel efforts in functional assays to simultaneously observe the nucleotide state of the active site and the position of the lever have the potential to provide the most important breakthroughs. Indeed, this kind of experiment has been accomplished previously by tracking the occupancy of ADP in myosin’s active site while simultaneously observing the mechanics of the power stroke (Ishijima et al., 1998). Thus, it may be possible to monitor when P_i_ is in the active site while also directly observing the rotation of the lever arm in a laser trap assay. This information will be vital for the development of new pharmaceutical compounds designed to gain control over the force-generating capacity of various classes of myosins to treat a myriad of myosin-related diseases.
